# Circulating levels of irisin in middle-aged first-degree relatives of type 2 diabetes mellitus — correlation with pancreatic β-cell function

**DOI:** 10.1186/1758-5996-6-133

**Published:** 2014-12-05

**Authors:** Meili Yang, Peihong Chen, Hua Jin, Xinmiao Xie, Ting Gao, Lili Yang, Xuemei Yu

**Affiliations:** Department of endocrinology and Metabolism, Fengxian Central Hospital, Shanghai, China; Department of endocrinology and Metabolism, The First Clinical Medical College of Suzhou University, Suzhou, China

**Keywords:** Type 2 diabetes mellitus, First-degree relatives, Irisin, Pancreatic β-cell

## Abstract

**Background:**

Irisin is a novel myokine secreted in response to peroxisome proliferator-activated receptor γ coactivator-1α (PGC-1α) activation through exercise. The first-degree relatives (FDRs) of type 2 diabetes mellitus (T2DM) patients bear a lifetime risk for developing T2DM, especially after 40 years old. However, the circulating irisin levels in middle-aged FDRs of T2DM is unclear. We therefore investigated the association between circulating irisin and pancreatic β-cell function in normal-glucose-tolerance (NGT) subjects.

**Methods:**

In this cross-sectional study, we recruited 412 supposed healthy subjects aged 40-60 who were FDRs of T2DM patients but without previous diagnosis of T2DM. Of the 412 individuals, 254 had NGT and 60 were newly diagnosed T2DM based on the results of a 75 g oral glucose tolerance test (OGTT- World Health Organization diagnostic criteria). We measured irisin in the newly diagnosed T2DM group (n = 60) and in an age- and sex-matched NGT subgroups (n = 62). Serum irisin was quantified by ELISA, and its association with metabolic parameters was analysed by Pearson’s correlation and multiple linear regression analyses.

**Results:**

There was no significant difference in serum irisin between middle-aged newly diagnosed T2DM patients and the NGT control group. Circulating irisin was correlated with haemoglobin A1c (r = 0.202, *p* = 0.026) and estimated glomerular filtration rate (r = 0.239, *p* = 0.010). Multiple linear regression revealed that only homeostasis model assessment-β (HOMA-β) was associated with irisin in NGT subjects after adjusting for confounding factors. However, similar analysis in T2DM did not reveal a significant association between circulating irisin and metabolic parameters.

**Conclusions:**

There was no significant difference in serum irisin between middle-aged newly diagnosed T2DM patients and the NGT controls. Serum irisin level was closely related to HOMA-β in NGT, suggesting that irisin may play a crucial role in pancreatic β-cell function.

## Background

Type 2 diabetes mellitus (T2DM) is a complex disease affected by many genetic and environmental factors. Genetic variants currently associated with susceptibility to T2DM only explain up to 10% of the putative “primary” contribution to T2DM risk [1]. It has been estimated that the susceptibility to develop T2DM in offspring of a T2DM parent is 3–4 times higher compared to the population without a family history of T2DM [2, 3]. Indeed, significant defects of insulin sensitivity, as well as of β-cell dysfunction, are present decades before first-degree relatives (FDRs) of T2DM develop the disease [4, 5]. The FDRs of T2DM bear a lifetime risk for developing T2DM, especially after 40 years old.

Skeletal muscle is an endocrine organ that produces and releases cytokines that have been named myokines [6–8]. Myokines work as endocrine hormones and regulate the functions of distant organs. Irisin, the proteolytic fibronectin type III domain-containing 5 (FNDC5)-cleaved product, upregulates peroxisome proliferator-activated receptor γ coactivator-1 α (PGC-1α) in rodent skeletal muscle. Irisin increases total energy expenditure and improves glucose tolerance in mice [9]. FNDC5 gene expression is positively and strongly correlated with the expression of PGC1-α in human muscle [10]. Although clear-cut data have been reported in rodents, the effect of irisin in humans remains controversial. Irisin level is inversely associated with intrahepatic triglyceride content in obese adults [11]. Irisin is thought to influence glucose homeostasis, based on the finding of decreased serum irisin in a cohort of drug-naive patients with new-onset T2DM compared to controls with normal glucose tolerance (NGT) matched for age, sex and body mass index (BMI) [12] and on the observation of lower circulating irisin in T2DM patients with the drug intervention compared with nondiabetic controls [13]. Accordingly, circulating irisin has shown a positive correlation with insulin sensitivity in a cohort of male subjects without T2DM [14], suggesting a beneficial role of irisin in glucose homeostasis. However, in a cohort of 118 patients, FNDC5 mRNA expression in muscle was not correlated to glucose homeostasis [15]. We therefore aimed to determine the circulating irisin levels between middle-aged newly diagnosed T2DM patients and the NGT controls of FDRs.

Exercise decreases inflammation and oxidative stress and improves the quality of life in patients with metabolic syndrome. Physical exercise enhances peripheral insulin sensitivity and favours β-cell growth and survival, so it is a powerful strategy to prevent and control diabetes [16–21]. On the basis of exercise’s effects on β-cell function, we explored the potential impact on β-cell function of irisin in NGT subjects.

## Materials and methods

### Recruitment and eligibility

The study was approved by the Ethics Committee of the Fengxian Central Hospital of Shanghai, China. All subjects provided written informed consent.

From December 2012 to September 2013, a community-based health survey was performed in the Fengxian District of Shanghai, China. A total of 412 FDRs of T2DM (192 men and 220 women), aged 40-60 years, were recruited. None had been diagnosed with T2DM. In addition, we evaluated the confounders variables such as eating, exercise habits, functional capacity from the questionnaire survey. All of them successively underwent a full evaluation of glucose tolerance status. Of the 412 individuals, 60 were newly diagnosed with T2DM, and 254 were NGT. We measured irisin in the newly diagnosed T2DM group (n = 60) and in an age- and sex-matched NGT group (n = 62). The assignment of subjects to NGT or T2DM groups was based on the results of a 75 g oral glucose tolerance test (OGTT) according to the World Health Organization diagnostic criteria (1999). Subjects with fasting plasma glucose (FPG) ≥ 7.0 mmol/L and 2h post-load plasma glucose (2h PG) ≥ 11.1 mmol/L were diagnosed with T2DM. Subjects with impaired fasting glucose and/or impaired glucose tolerance were excluded. Individuals with a history of T2DM, gestational diabetes, acute or chronic inflammatory disorders, cancer, active hepatitis/liver cirrhosis, severe cardiovascular or kidney diseases or other known major disease were precluded from the study.

### Anthropometric and biochemical measurement

Weight (without shoes and in light outdoor clothing) and height were measured, and BMI (kg/m^2^) was calculated by dividing weight (kg) by height^2^ (m^2^). The subjects stood with their feet separated by 25 cm to 30 cm to evenly distribute their weight. Waist circumference was measured midway between the lower rib margin and the iliac crest at the end of expiration, hip circumference measurement was around the most prominent point of hip pelvis. All blood samples were taken in the morning following an overnight fast of at least 8 h and immediately centrifuged. A 75 g glucose was administered orally, and plasma glucose was measured in 2 hrs. In all subjects, FPG, 2h PG, serum total cholesterol(TC), triglycerides(TG), low-density lipoprotein cholesterol (LDL-C), high-density lipoprotein cholesterol (HDL-C), free fatty acids (FFA), alanine transaminase (ALT), aspartate transaminase (AST), blood urea, serum creatinine, and uric acid (UA) were measured using an automatic biochemical analyser (Beckman DXC800, USA). Haemoglobin A1c(HbA1c) was measured using a fully automatic glycosylated haemoglobin analyser (TOSOHHLC-723G7, Japan). Fasting insulin (FINS) was measured by electrochemiluminescence immunoassay (BECK, Germany). Homeostasis model assessment of insulin resistance (HOMA-IR) values were calculated as FPG(mM) × FINS (mU)/22.5. Homeostasis model assessment-β (HOMA-β) values were calculated as 20 × FINS (mU)/[FPG (mM)- 3.5]. Skeletal muscle, body fat mass, body fat percentage were measured by human body composition analyser (INBODY S10, Korea). Estimated glomerular filtration rate (eGFR) was estimated from calibrated serum creatinine values using the Modification of Diet in Renal Disease (MDRD) study equation as follows: eGFR (ml/min/1.73m^2^) = 175 × (serum creatinine^-1.154^) × (age^-0.203^) × 0.742 (if female). Serum irisin levels were determined using a commercially available human ELISA kit (Phoenix Pharmaceutics, Inc, USA).

### Statistical analysis

Statistical analysis was performed using SPSS 17.0 software. The data are presented as the mean ± SD. Student’s *t* test was employed to compare the means of normally distributed continuous variables between two groups. Categorical data were compared by the χ^2^ test. Pearson’s correlation analysis was performed to study the correlation between two continuous variables. Multiple linear regression models were employed to identify variables that were independently associated with irisin concentration. *P* < 0.05 was considered statistically significant.

## Results

### Characteristics of study participants

The clinical and laboratory characteristics of the study subjects are presented in Table 1. Age, sex, hip circumference, TC, HDL-C, LDL-C, blood urea, serum creatinine, skeletal muscle, body fat percentage, and irisin were not significantly different between NGT and T2DM subjects. T2DM subjects had higher waist circumference, waist-to-hip ratio, systolic blood pressure, diastolic blood pressure, BMI, TG, FPG, 2h PG, HbA1C, insulin, HOMA-IR, FFA, ALT, AST, UA, eGFR, and body fat mass compared with NGT subjects. HOMA-β was lower in T2DM compared with NGT subjects. But, systolic blood pressure, diastolic blood pressure, ALT, AST, UA, and FFA were normal in both groups. No significance was observed on the life-style questionnaire included eating, exercise habits and functional capacity between NGT and T2DM subjects.

**Table 1:** **Baseline clinical characteristics of subjects with NGT and T2DM**

	NGT	T2DM	***P***value
n	62	60	
Age (years)	52.03±5.59	51.81±5.77	0.837
Sex (M/F)	28/34	29/31	0.446
Waist (cm)	80.55±9.06	87.41±8.55	<0.001
Hip (cm)	91.40±5.58	93.26±6.87	0.106
WHR	0.88±0.08	0.94±0.08	<0.001
BMI (kg/m^2^)	24.07±3.21	25.80±2.99	0.003
Systolic BP (mmHg)	121.53±13.97	134.17±16.14	<0.001
Diastolic BP (mmHg)	78.68±9.00	84.66±10.12	0.001
TC (mM)	5.27±1.26	5.43±1.09	0.47
TG (mM)	1.25±0.66	2.27±1.77	<0.001
HDL-C (mM)	1.31±0.29	1.32±0.24	0.901
LDL -C(mM)	3.11±0.88	3.14±0.80	0.837
FFA (mM)	0.35±0.14	0.59±0.24	<0.001
FPG (mM)	5.13±0.47	7.80±2.50	<0.001
2h PG (mM)	5.84±1.10	15.61±4.34	<0.001
HbA1C (%)	5.50±0.34	7.00±1.78	<0.001
Insulin (mU)	6.42±3.62	9.68±4.17	<0.001
HOMA-β	82.63±47.49	61.25±43.16	0.011
HOMA-IR	1.48±0.90	3.20±1.33	<0.001
ALT (IU/L)	25.03±10.88	36.27±24.28	0.002
AST (IU/L)	22.73±5.34	27.23±14.41	0.026
Urea (mM)	4.67±1.21	4.76±0.98	0.674
Creatinine (μM)	62.26±14.04	58.17±13.87	0.108
Uric acid (μM)	258.81±76.83	301.57±76.99	0.003
eGFR (ml/min/1.73 m^2^)	104.73±21.13	117.40±31.12	0.011
Muscle mass (kg)	23.42±5.08	24.38±4.59	0.286
Fat mass (kg)	19.29±6.01	22.43±5.41	0.004
Percentage of body fat (%)	30.92±6.88	33.25±5.88	0.052
Irisin (ng/ml)	277.62±131.07	289.88 ±141.00	0.619

Data are expressed as the mean ± SD.

WHR: waist-to-hip ratio; BMI: body mass index; BP: blood pressure; TC: total cholesterol; TG: triglyceride; HDL-C: high-density lipoprotein cholesterol; LDL-C: low-density lipoprotein cholesterol; FFA: free fatty acids; FPG: fasting plasma glucose; 2h PG: 2h plasma glucose; HbA1C: haemoglobin A1C; HOMA-β: homeostasis model assessment-β; HOMA-IR: homeostasis model assessment of insulin resistance; ALT: alanine transaminase; AST: aspartate transaminase; eGFR: estimated glomerular filtration rate.

### Correlation of irisin with clinical parameters

**Figure 1:**
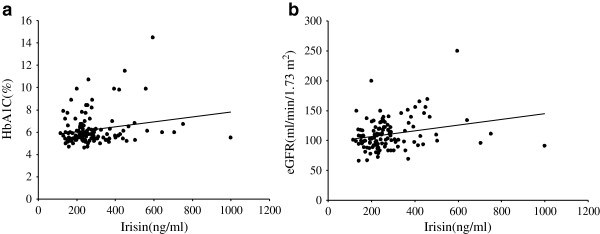
**The correlation analysis between HbA1C and eGFR and circulating irisin.** Pearson bivariate correlation analysis showed that HbA1C (r = 0.202, p = 0.026) and eGFR (r = 0.239, p = 0.010) were positively correlated with circulating irisin, the figures were presented in **a**, **b**.

Correlation coefficients between irisin level and the measured clinical parameters in two groups were presented in Table 2. Pearson bivariate correlation analysis showed that HbA1C (r = 0.202, *p* = 0.026) and eGFR (r = 0.239, *p* = 0.010) were positively correlated with circulating irisin, and revealed no correlation between irisin and other metabolic parameters. The figures of the correlation analysis between HbA1C and eGFR and circulating irisin are presented in Figure 1a, b.

**Table 2:** **Pearson correlation coefficients between irisin and metabolic parameters**

	***r***	***P***value
Age (years)	-0.073	0.434
Waist (cm)	0.064	0.491
Hip (cm)	0.165	0.071
WHR	-0.043	0.64
BMI (kg/m^2^)	0.08	0.383
Systolic BP (mmHg)	0.016	0.864
Diastolic BP (mmHg)	-0.079	0.389
TC (mM)	0.088	0.338
TG (mM)	0.144	0.115
HDL-C (mM)	0.075	0.409
LDL-C (mM)	0.071	0.439
FFA (mM)	0.06	0.52
FPG (mM)	0.165	0.069
2h PG (mM)	0.077	0.399
HbA1C (%)	0.202	**0.026**
Insulin (mU)	0.166	0.069
HOMA-β	0.161	0.079
HOMA-IR	0.178	0.052
ALT (IU/L)	0.001	0.991
AST (IU/L)	-0.063	0.489
Urea (mM)	-0.127	0.165
Creatinine (μM)	-0.126	0.166
Uric acid (μM)	0.047	0.609
eGFR (ml/min/1.73 m^2^)	0.239	**0.01**
Muscle mass (kg)	0.019	0.841
Fat mass (kg)	0.018	0.85
Percentage of body fat (%)	0.01	0.914

WHR: waist-to-hip ratio; BMI: body mass index; BP: blood pressure; TC: total cholesterol; TG: triglycerides; HDL-C: high-density lipoprotein cholesterol; LDL-C: low-density lipoprotein cholesterol; FFA: free fatty acids; FPG: fasting plasma glucose; 2h PG: 2h plasma glucose; HbA1C: haemoglobin A1C; HOMA-β: homeostasis model assessment-β; HOMA-IR: homeostasis model assessment of insulin resistance; ALT: alanine transaminase; AST: aspartate transaminase; eGFR: estimated glomerular filtration rate. *P* values < 0.05 were shown in bold.

As shown in Table 3, among the independent variables included in the multiple linear regression model, HOMA-β was independently associated with irisin level after adjusting for other co-variables in NGT controls, but not in new T2DM patients. Multiple linear regression also revealed that irisin was correlated with HOMA-β (β = 1.872, *p* = 0.025) and FPG (β = 1.012, *p* < 0.001) in NGT after adjusting for 2h PG, HbA1C, insulin, and HOMA-IR.

**Table 3:** **Association of irisin with HOMA-β in fully adjusted model**

	NGT		T2DM	
	***β***	***P value***	***β***	***P value***
Model 1	0.3	**0.02**	0.053	0.688
Model 2	0.345	**0.016**	0.006	0.971
Model 3	0.41	**0.006**	0.056	0.693
Model 4	0.349	**0.012**	0.069	0.642
Model 5	1.872	**0.025**	-0.039	0.926

Model 1: unadjusted. Model 2: adjusted for ALT, AST, urea, creatinine, uric acid, and eGFR. Model 3: adjusted for waist circumference, hip circumference, WHR, BMI, muscle mass, fat mass, and percentage body fat. Model 4: adjusted for TC, TG, HDL-C, LDL-C, and FFA. Model 5: adjusted for 2h PG, HbA1C, insulin, and HOMA-IR.

*P* values < 0.05 were shown in bold.

## Discussion

Our result that serum irisin level in NGT FDRs was closely related to HOMA-β (Table 3) suggests that irisin may play a crucial role in pancreatic β-cell function. Obesity-induced inflammation in pancreatic tissue promotes apoptosis of β-cells and results in insulin deficiency [22, 23]. Irisin could contribute to the modulation of obesity-induced inflammatory/anti-inflammatory balance by increasing CD206 and interleukin 10 and decreasing tumour necrosis factor alpha and leptin [14]. Irisin is an exercise-induced hormone secreted by skeletal muscle that can drive brown-fat-like conversion of white adipose tissue. This effect is possibly mediated by irisin-induced phosphorylation of p38 mitogen-activated protein kinase (p38 MAPK) and the extracellular signal-regulated protein kinase (ERK) signalling pathway to prevent obesity and T2DM. Furthermore, irisin promotes the expression of betatrophin, another newly identified hormone that promotes pancreatic β-cell proliferation and improves glucose tolerance [24]. So, we speculate that irisin promotes insulin secretion by increasing the proliferation or reducing the apoptosis of β-cells. The mechanism underlying the correlation of irisin with β-cell function in NGT is unknown. Due to the limited sample size, we did not find such a correlation in T2DM patients.

Peroxisome proliferator-activated receptor gamma coactivator-1-alpha (PGC-1α) is a versatile transcription cofactor involved in glucose/fatty acid metabolism, insulin secretion, and mitochondrial function in liver, pancreas, adipocytes and muscle. Irisin secretion after PGC1α activation in response to exercise could enhance insulin sensitivity and increase energy expenditure in animal experiments [25–28], which has also been proposed link physical activity and energy metabolism [29, 30]. In our study, there was no significant difference in serum irisin between middle-aged newly diagnosed T2DM patients and the NGT control group, which was presumably related to impaired muscle PGC-1α expression among these FDRs of T2DM patients. Our conjecture is in general consistent with what Liu et al [13] previously reported: lower irisin was secondary to impaired PGC-1α expression and function in muscle in T2DM. Irisin is produced within muscle, and total muscle volume can affect the irisin level. Stengel et al [31] found that circulating irisin was affected under conditions of altered BMI, with the highest levels in severely obese patients, while the altered BMI levels of our study were very small. Al-Daghri et al [32] reported that circulating irisin level was negatively correlated with HOMA-IR in a cross-sectional study of girls aged 12.9 ± 3.2 years-old, suggesting that irisin secretion at an early age might delay the onset of obesity, insulin resistance and T2DM. Thus, for middle-aged NGT FDRs of T2DM patients, promoting irisin secretion through exercise at an early age might be equally important as to newly diagnosed T2DM patients.

To avoid the potential effect of genetic polymorphism, hypoglycaemic agents and diabetic complications, our study population was FDRs without a definitive diagnosis of diabetes living in Fengxian, Shanghai, China. We attempted to analyse the association between circulating irisin and markers of metabolic phenotypes to elucidate the potential role of irisin in energy metabolism in humans. Correlation analysis revealed that circulating irisin level was positively correlated with HbA1C (r = 0.202, *p* = 0.026) and eGFR (r = 0.239, *p* = 0.010). Choi et al [12] found that irisin was negatively correlated with HbA1C. Guo et al [33] reported that HbA1c varied as a function of age and race, and HbA1c had low sensitivity and high specificity for identifying diabetes when assessed against diagnoses using both FPG and 2h PG. Therefore, our finding of an opposite correlation of irisin with HbA1C level was most likely a result of the different age, race, and geographic variation, which also may be related to different stages of diabetes of the patient populations. Wen et al [34] observed that the decrease in irisin in chronic kidney disease patients was inversely correlated with the level of creatinine, which was consistent with our results. Further therapeutic clinical trials or animal studies are necessary to clarify the mechanisms underlying the effects of irisin on renal function. It is also important to test if the irisin level correlates with the progression of diabetic nephropathy.

Circulating irisin level showed no correlation with BMI, which was in keeping with Timmons et al [15], who reported that myocyte expression of irisin was not related to markers of energy metabolism, including BMI. Choi et al [12] found that irisin was negatively correlated with BMI, which was opposite to the findings of Huh et al [10] and Liu et al [13]. We think that these discrepancies may be explained by the differences in their study populations and exercise habits.

In our study, multiple linear regression revealed that irisin was associated with FPG (β = 1.012, *p* < 0.001) in NGT subjects after adjusting for 2h PG, HbA1C, insulin, and HOMA-IR. This finding is in general consistent with Huh et al [10] and Liu et al [13]. All of these findings indicate that irisin is closely associated with glucose concentration, especially FPG, but the causal relationship is still not clear.

There were limitations to the current study. First, our sample size was relatively small, and the age range of subjects was relatively limited. Second, we did not measure the change in insulin secretion pattern or the proportion of proinsulin and insulin. Third, the NGT subjects but not FDRs of T2DM were not included in this study, and the expression of *PGC1-α*/*PPARGC1* was decreased inT2DM and in high-risk non-diabetic subjects with a family history of T2DM [35], giving to the present study an important limitation to assure the conclusions. But, in our study, there was no significant difference in muscle mass and physical activity between newly diagnosed T2DM patients and the NGT control group. Though the current data do not support the concept of irisin being induced by exercise, in contrast to Timmons et al [15], Raschkeet al [36], Erickson et al [37], and Hofman et al [38], making it less likely that the beneficial effect of irisin observed in mice can be translated to humans, we appreciate that small cohorts of different individuals may display different results. In fact, both Boströmet al [9] and Timmonset al [15] found increased FNDC5 mRNA levels with exercise, at least in older subjects. In addition, analyses of genomic DNA, mRNA and expressed sequence tags have revealed that human FNDC5, is sometimes mutated in the conserved start codon from ATG to ATA [36]. Future studies should address the FNDC5/irisin expression and cleavage mechanisms to resolve the current controversy.

## Conclusion

In summary, there was no significant difference in serum irisin between middle-aged newly diagnosed T2DM patients and the NGT control group. Multiple linear regression model revealed that HOMA-β was associated with irisin in NGT subjects after adjusting for multiple co-variates, suggesting that irisin may play a crucial role in pancreatic β-cell function. Further studies are needed to study the relationship between irisin and pancreatic β-cell function and the molecular mechanisms of its role in insulin secretion.

## Abbreviations

FDR: First-degree relative
T2DM: Type 2 diabetes mellitus
NGT: Normal glucose tolerance
FNDC5: Fibronectin type III domain-containing 5
PGC-1α: Peroxisome proliferator-activated receptor γ coactivator-1 α
WHR: Waist-to-hip ratio
BMI: Body mass index
TC: Total cholesterol
TG: Triglycerides
HDL-C: High-density lipoprotein cholesterol
LDL-C: Low-density lipoprotein cholesterol
FPG: Fasting plasma glucose
2h PG: 2h post-load plasma glucose
HbA1C: Haemoglobin A1C
HOMA-β: Homeostasis model assessment-β
HOMA-IR: Homeostasis model assessment of insulin resistance
FFA: Free fatty acids
ALT: Alanine transaminase
AST: Aspartate transaminase
eGFR: Estimated glomerular filtration rate
MAPK: Mitogen-activated protein kinase
ERK: Extracellular signal-regulated kinase
FINS: Fasting insulin
UA: Uric acid.
